# Chronic-pharma: New Platform for Chronic Patients Pharmacotherapy Optimization

**DOI:** 10.1007/s10916-022-01808-0

**Published:** 2022-02-28

**Authors:** Angela María Villalba-Moreno, Mercedes Galván-Banqueri, Aitana Rodríguez-Pérez, María Dolores Toscano-Guzmán, Clara López-Hermoso, Susana Sánchez-Fidalgo, Bernardo Santos-Ramos, Eva Rocío Alfaro-Lara

**Affiliations:** 1 Pharmacy Service, University Hospital Virgen del Rocío, Seville, Spain; 2 Pharmacy Service, University Hospital Virgen de Valme, Seville, Spain; 3 Department of Preventive Medicine and Public Health, University of Seville, Seville, Spain

**Keywords:** Clinical decision support system, Technological development, Chronic patient, Deprescribing, Trigger tool, Anticholinergic burden

## Abstract

We describe the technological development of a web platform named CHRONIC-PHARMA that integrates three prescription support tools for patients with chronic diseases: Anticholinergic Burden Calculator (ABC), LESS-CHRON criteria and TRIGGER-CHRON. They focus on the optimization and evaluation of pharmacotherapy in patients with chronic diseases, resulting in a useful, single platform that can facilitate the review of pharmacotherapy and improve the safety of chronically ill patients. This is achieved by estimating and reducing the anticholinergic risk (ABC), detecting opportunities for deprescribing drugs and monitoring its success (LESS-CHRON criteria), as well as calculating the risk of adverse drug events (TRIGGER-CHRON). The platform is freely accessible online (https://chronic-pharma.com/) as well as through a mobile application, and therefore easily accessible among the healthcare community.

## Introduction

The high prevalence of chronic diseases is changing the profile of patients who require healthcare. Patients with chronic diseases are a group of special interest since their care is predominated by complex polypharmacy, which is a prime risk factor for inappropriate prescribing and adverse drug reactions and events. Implementing an appropriate treatment plan for these patients is a difficult and complex process. They require a comprehensive quality approach, with appropriate pharmacotherapeutic optimization mediated by prescription support tools which standardize the recommendations of pharmacotherapy, considering the specific situation of the patient [1]*.* Creating such prescription support tools is an additional challenge; they need to be designed, built, validated, and tested robustly so that they can help to perform complex medication reviews.

There are widely accepted tools that have been consolidated as guidelines to help in the global review of treatment in elderly patients: STOPP-START criteria or the Beers criteria [2, 3]. They are fundamental tools to be considered in any digital platform for the global review of treatment in elderly patients. Indeed, a computer-generated STOPP/START is currently being investigated to examine the impact of criteria on incident ADRs (SENATOR) and drug-related hospitalizations (OPERAM) [4].

In recent years, other tools have been published that have also added value to the pharmacotherapeutic review process from more specific perspectives in complex chronic patients. This is the case of anticholinergic scales or deprescribing strategies, between others. In this case, the development of its concepts has often gone hand in hand with its technological implementation, since the advantage of electronic prescription support systems in the medication review process has been demonstrated [5, 6].

The risk of developing adverse anticholinergic effects is a significant issue in polypharmacy. Tools to identify this risk are named anticholinergic scales [7, 8]. Our research group (http://chronicpharma.es/) has developed a tool which calculates the anticholinergic burden based on ten anticholinergic scales. The scales have been firstly identified in a systematic review carried out in chronic patients with multimorbidity, with the aim to be used in clinical settings [9]. The tool was named the Anticholinergic Burden Calculator (ABC), and accessible online via a web portal software program (www.anticholinergicscales.es/) [10].

In recent years, the concept of deprescribing has been raised worldwide as an essential review process to reduce the therapeutic burden (drug burden) [11, 12]. Some guidelines are aimed at deprescribing specific groups of drugs such as benzodiazepines or proton pump inhibitors (for example, Canadian deprescribing guidelines) [13, 14], or to identify patients whose treatments contain drugs to be deprescribed as suggested by deprescribing criteria focused on different type of patients [15, 16]. The LESS-CHRON criteria (List of Evidence baSed depreScribing for CHRONic patients) are a comprehensive and standardized methodology to identify clinical situations for deprescribing drugs in chronic patients with multimorbidity, considering life prognosis and providing health variables to value the safety of the process. They were translated into English using a transcultural methodology [17], and validated by a reliability study [18].

The trigger tool methodology was developed by the Institute for Healthcare Improvement as a low-resource option to detect adverse events within hospital settings [19]. Trigger tools involve the application of various screening criteria to guide the medical review process in the identification of adverse events [20]. They seem to be the most efficient and cost-effective singular method for detecting harm associated with health care, which is why numerous studies using the trigger method to measure adverse drug events (ADEs) rates in health care organizations have been published [21]. TRIGGER-CHRON has recently been developed as a tool that uses the trigger method for elderly patients with multimorbidity. The development was carried out in two consecutives phases. Firstly, the most common triggers for that population were selected [22]. Secondly, 32 of that 51 previously identified, were selected based on their positive predictive value, obtaining the TRIGGER-CHRON [23].

Healthcare is facing the emergence of a new range of systems and applications using electronic communication. The use of health applications has recently seen rapid and steady growth. In 2014, compared to the previous year, the time spent on applications in the health category increased by 89% [24].

Thus, the objective of this work is to describe the technological development of a platform that integrates the three tools developed by our research group: the ABC Calculator, the LESS-CHRON Criteria and the TRIGGER-CHRON criteria.

## Methods and study design

The web platform and mobile app development was conducted from November 2018 to June 2020.

**Fig. 1:**
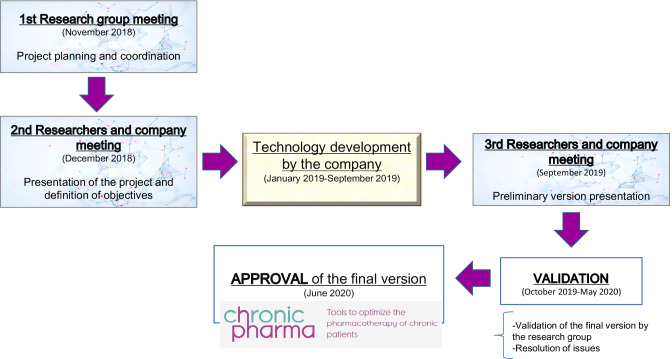
Diagram of the evolution of the project phases “*CHRONIC-PHARMA*” web

Previously, each of the tools (ABC, LESS-CHRON and TRIGGER-CHRON) were developed within a multidisciplinary team of hospital pharmacists, internal medicine specialists and general practitioners [10, 17, 22]. To integrate the knowledge generated into the web format, a research group was formed with the hospital pharmacists responsible for each tool, a total of six professionals. An external company with experience in transferring research results to the market was contracted. The research group directed and supervised the company in the design of the website and app, the development of the content and the prototype, ensuring that the development of the platform met the objectives established. Finally, the researchers conducted the usability evaluation of the provisional version before completing the process (Fig. 1).

The design and visual appearance of the app was the same as that used for the web platform. It was developed for the Android platform.

In order to validate the usability, each investigator responsible for the tool performed separate performance tests on simulated patients with multiple scenarios, followed by a second investigator. Finally, the research group reviewed the final version for enhancements and bug corrections.

## Results

**Fig. 2:**
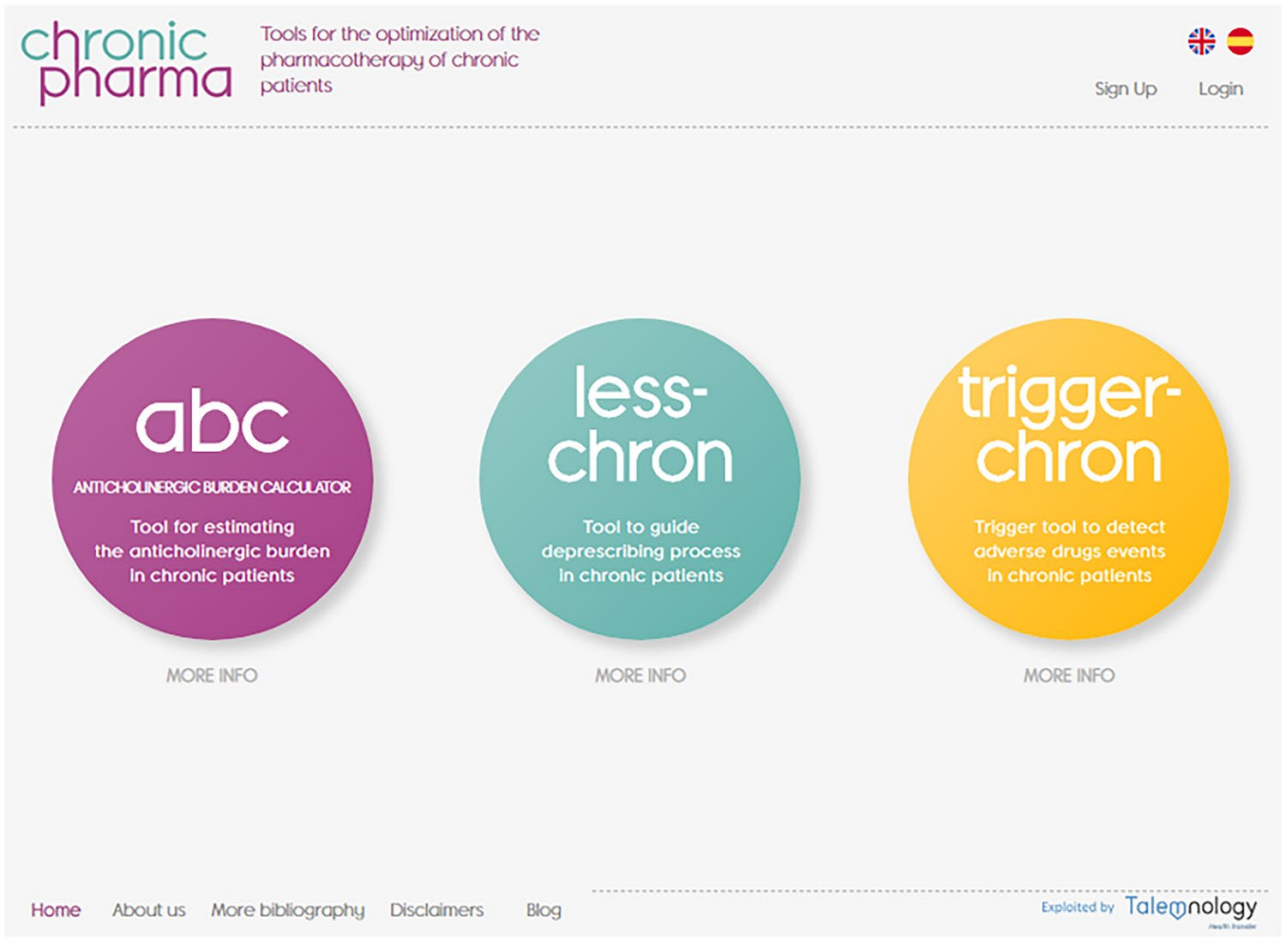
Homepage of *CHRONIC-PHARMA*-web

The platform created is called CHRONIC-PHARMA. It is available via a web page (https://chronic-pharma.com/) and a mobile health application available on the Google Play Store. It has integrated three tools that can be used independently in each registered patient: estimating the anticholinergic risk and assessing possible interventions for its reduction (ABC), detecting drugs to be deprescribed and monitoring its success (LESS-CHRON) and calculating the risk that a patient has of having an ADE (TRIGGER-CHRON). (Fig. 2). All three have been developed using robust systematic review methodologies and structured clinical consensus [10, 17, 22]. ABC includes previously published and validated scales identified in a systematic review [9]. LESS-CHRON and final TRIGGER-CHRON are in the process of validation in patients, which is the most expected study for a tool of this category; however, both has been applied in a theorical way, validating its utility [18, 23]. In relation to the complexity when adapting the tools to web format, it should be noted that ABC differs from the LESS-CHRON and TRIGGER-CHRON tools in the modus operandi it requires, as well as in the expression of the results. For example, DBI requires a calculation considering daily dosage of drugs [25], LESS-CHRON and TRIGGER-CHRON serve as a guide for the preparation of a final recommendation report that does not establish weight differences between the prescribed drugs, but it adheres to pre-specified criteria that the user must decide if they are met or not in the target patient.

The web/app platform is free access and can be used in both English and Spanish. We therefore hope that this platform can be disseminated and used by healthcare professionals at any level of care. The platform structure is simple, with users filling in the data that it asks for depending on the characteristics and pharmacotherapy of each patient, and not having to review each of the criteria or drugs.

Each tool is accompanied with an explanation on how to use it, as well as its interpretation.

**Fig. 3:**
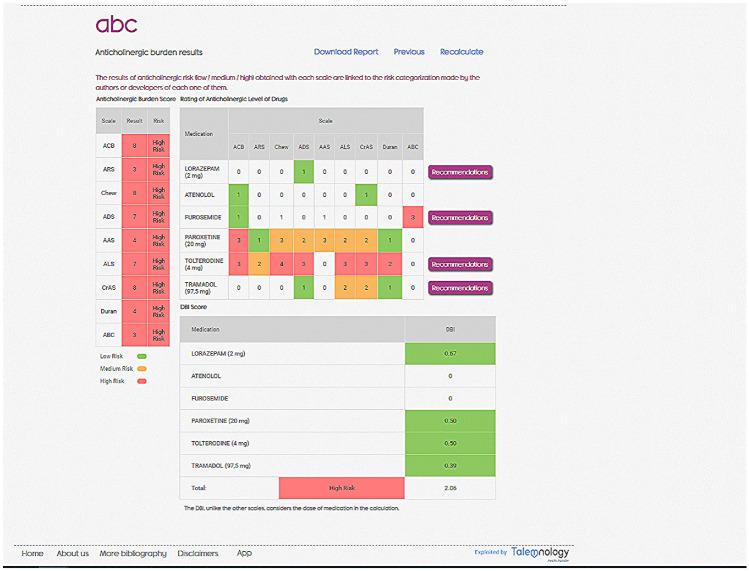
Example of a result with the ABC Calculator

**Fig. 4:**
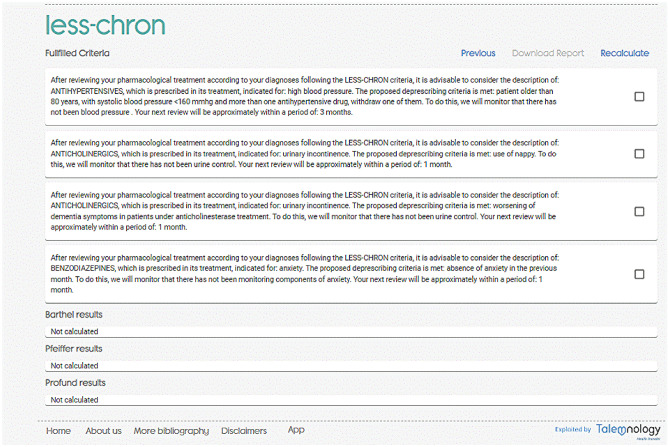
Example of report with the LESS-CHRON tool

**Fig. 5:**
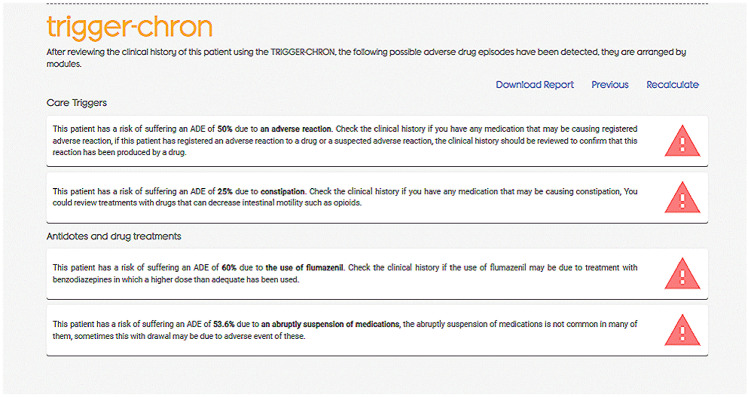
Example of triggers with the TRIGGER-CHRON tool

1. **ABC.** The anticholinergic burden is calculated based on the nine anticholinergic scales and Drug Burden Index (DBI), after introducing anticholinergic drugs and the total daily dose required for DBI. The burden is reported quantitatively and the risk for each scale is qualitatively color-coded (null, low, medium, and high). The individual drugs involved are also reported along with their anticholinergic potential given by each scale. Due to the variability among the anticholinergic scales, ABC calculator is intended to be an aid to the calculation by offering all the possibilities. However, a global interpretation is recommended, and the professional must decide regarding the clinical context of the patient. For example, furosemide is considered anticholinergic in four of the scales included in ABC, however, in three of them the potential is low or null. Furosemide is a frequently prescribed drug, so it is not appropriate to consider that it is the cause of the anticholinergic risk individually. If the total load is high, it could be useful to optimize treatment by intervening in furosemide due to the cumulative effect of anticholinergic drugs. On the other hand, suggestions for changes in treatment are included to reduce anticholinergic burden. From this, an accurate and up-to-date report of suggestions on pharmacological treatment to reduce the risk of anticholinergic events can be generated for each patient (Fig. 3).
2. **LESS-CHRON.** This tool follows the structure of the LESS-CHRON, initially asking users to select the therapeutic groups included in patient treatment (cardiovascular, genitourinary, etc.). This reduces the options and then only shows criteria that applies specifically to the patient if the conditions are met. Some LESS-CHRON criteria require understanding the patient’s cognitive function (according to the Pfeiffer questionnaire [26]), functional function (according to the Barthel index [27]) and / or prognosis (according to the Profund Index [28]). The LESS-CHRON tool incorporates these indexes and can calculate them instantly if needed. Finally, a recommendation report is obtained that includes the medications to be deprescribed, as well as a proposal for the review date of patients (which is included in the LESS-CHRON Agenda) and the health variables to be monitored. Example of a recommendation report in the LESS-CHRON criteria is shown in Fig. 4.
3. **TRIGGER-CHRON.** This tool supports users to carry out a guided review of the patient's medical records, explore different situations or risks that could occur to each patient. These situations, or triggers, are organized in 5 modules: care, antidotes and treatment, plasma concentration, analytical parameters and emergencies., These are shown progressively, and the user should click/mark those that have occurred in the patient. Finally, the tool calculates, based on the positive predictive value, the risk that the patient has of suffering adverse drug events. It also highlights the medication or medications that may be responsible (Fig. 5).

## Discussion

CHRONIC-PHARMA is the result of an innovative research process focused on chronic patients, whose results have been transformed into practical value for clinical practice. The integration of these three tools in a single web/app platform has improved the accessibility of professional support tools. It is one of the most comprehensive web/app platforms currently available that focuses on the optimization and evaluation of pharmacotherapy in a population of chronic patients.

There are tools that have already been published in the literature to support prescriptions aimed at the same type of patients. This is the case of the PRIMA-eDS project and the G-MEDSS tool, between others. These differ from the CHRONIC-PHARMA tool in their nature. Thus, PRIMA-eDS, apart from being a tool designed to be integrated in a computerized decision support system, is being developed in the context of a randomized clinical trial and consists of a database that allows analysis of patient prescriptions, issuing recommendations [29]. On the other hand, G-MEDSS was developed based on the methodology of a previous tool, DBI calculator, expanding its usefulness by adding other tools through a scenario-based design [5]. CHRONIC-PHARMA has not been validated as a platform, but rather each of the tools it integrates have been developed in different clinical contexts with rigorous methodologies for their creation and with the participation of potential users.

We intend to carry out activities that favor its implementation and visibility, such as its dissemination through clinical sessions, presence in national and international scientific conferences, as well as in scientific societies. Collaboration with clinicians is necessary to promote its use and the involvement of external biotechnology companies (public or private) who can support technical development, scalability, and dissemination of the platform. This is the reason why our research group is currently carrying out multicenter projects in which the tools included in the platform are used to support the review of medications by geriatricians, internists and pharmacists from hospitals, primary care and nursing homes. The main strength of this project is the development of an innovative platform that could be used by healthcare professionals to optimize the pharmacotherapy in this specific, yet ever-expanding group of patients.

CHRONIC-PHARMA is presented as a fast, simple, free access and attractive platform, available in Spanish and English, which translates into greater global visibility. It offers a single record of patient data as well as obtaining a final report of recommendations in each of the tools. It allows users to register the patients and save their information in a safe way (password access and encrypted patient data), useful for clinical practice and research.

We hope that the web/app will make it easier for prescribers to review the medication of chronic patients. Its implementation in clinical practice can help to quickly detect drugs that can be suspended or modified to reduce the risk of ADE, as well as to quickly detect drug-related adverse events. This would decrease the pharmacological burden in these patients, who usually take five drugs or more per day [30], and ultimately improve safety.

We consider that this platform is capable of being incorporated into clinical practice guidelines and protocols aimed at these patients. However, the platform is not currently planned to be integrated into the clinical history computer systems, as it is a complex process that requires more experience and institutional support.

The first version of ABC has already had great impact, with a total of 5,000 registrations to the platform, half of them registered in 2019. Users are based primarily in Spain, UK, USA, France, Australia and Canada. In 2019, 21,500 calculations were made with 13,110 new patient files and around 181,000 visits to the website. There have also been numerous publications that have referenced the tools that make up this platform [31–37]. In the most recent reviews on this topic, LESS-CHRON is referenced as a powerful tool to be used amongst the chronic patient [34, 35]. The quick and widespread acceptance of these tools encourages us to promote this platform more widely in the healthcare environment, as well as to continue developing our research work to implement new tools and update current ones.

The platform aims to be a portal that can be extended to incorporate further tools developed with a rigorous methodology focused on improving the safety and pharmacotherapy of the chronic patient, such as tools to improve compliance, review of adequacy, reconciliation, or drug-drug interactions. One limitation to this platform is that it is not capable of extracting the information from patient’s clinical history directly, and healthcare professionals must input the data manually.

As for the treatment modification recommendations included in ABC, are based on a systematic review carried out by the working group whose objective was to ascertain the available scientific evidence on actions carried out to reduce a high anticholinergic load in the elderly. The work to keep these recommendations updated is continuous and any changes to recommendations will be incorporated in the next update. Thanks to this new version, support in decision-making is offered to optimize treatment in relation to anticholinergic risk.

The platform can be applied systematically to chronic patients to detect drugs that present increased risk to patients, rather than benefits. However, to apply the LESS-CHRON criteria, we recommend complementing the results with a clinical interview. In some cases, it is also necessary to calculate other scales such as the Barthel Index or the Pfeiffer Scale. To simplify these calculations, access to these scales has been provided directly on the platform. In addition, the platform allows users to set alerts on the recommended date to review the success or failure of the prescription. The TRIGGER-CHRON tool is the only list of triggers for chronic and multimorbid elderly patients which allows identifying adverse drug event and their possible prevention. It has shown that from every 4 reviewed medical records an adverse drug event can be detected.

In terms of applicability, it is a platform that is useful at both hospital and primary care levels. In the case of a hospital admission, it should be a priority to use the TRIGGER-CHRON to detect patients at risk of suffering adverse drug events. Subsequently, it is also useful to apply the two remaining tools on anticholinergic burden and deprescription for the comprehensive assessment of pharmacotherapy of chronic patients at time of discharge. On the other hand, the systematic use of the platform in primary care could help in the review of polymedicated patients to reduce adverse events associated with medication. The benefits could be both short and long term. Its use is also extendable to physicians and pharmacists, among other health professionals. It is expected to have an impact not only on health by improving patient safety, but also at the organizational level, providing uniformity in decision making and clinical judgment. Finally, it is possible that the reduction of polytherapy in appropriate cases, will lead to a reduction in costs of the drugs and derived from possible medical visits or hospitalizations.

It is therefore expected that the CHRONIC-PHARMA platform will have a high acceptance by the scientific and healthcare community, as the combined use of the three tools through a user-friendly, digital interface is more comprehensive, efficient and easily accessible.

## Conclusions

In conclusion, the newly developed CHRONIC-PHARMA web and mobile app for chronic patients is a potentially useful platform in the modern technological era. CHRONIC-PHARMA integrates three useful tools into a single portal, for the optimization of pharmacotherapy of chronic patients.

The app is available in the Google Play store and both the website and app are free and can be shared and distributed among the healthcare community.

## Data Availability

Protected custom code.
